# Imipramine inhibits feline infectious peritonitis virus infection by targeting host cholesterol metabolism via NPC1

**DOI:** 10.1128/aac.01866-25

**Published:** 2026-06-23

**Authors:** Rui Jing, Yao Yao, Yuhan Li, Shuo Zheng, Hongxuan Li, Li Kang, Li Gao, Pinghuang Liu

**Affiliations:** 1 State Key Laboratory of Veterinary Public Health and Safety, College of Veterinary Medicine, China Agricultural University 630101 , Beijing, China; 2 Key Laboratory of Animal Epidemiology of the Ministry of Agriculture and Rural Affairs, College of Veterinary Medicine, China Agricultural University 630101 , Beijing, China; Chinese Academy of Medical Sciences & Peking Union Medical College, Beijing, China

**Keywords:** FIPV, imipramine, NPC1, cholesterol metabolism

## Abstract

Feline infectious peritonitis (FIP), a fatal disease caused by FIP virus (FIPV), currently lacks effective vaccines and treatments. Imipramine, a tricyclic antidepressant with known broad-spectrum antiviral properties, was investigated for its efficacy against FIPV. Our *in vitro* studies demonstrated that imipramine inhibits FIPV infection in a dose-dependent manner, with a high selectivity index (SI = 291) and maximal effect when administered during co-treatment. Time-of-addition assays revealed that imipramine acts at multiple stages, interfering with both viral entry and replication. Mechanistically, we found that imipramine targets the host protein Niemann-Pick C1 (NPC1), disrupting cholesterol metabolism to block viral entry. It also independently suppresses FIPV-induced pro-inflammatory cytokine production without relying on type I interferon signaling. Notably, in an *in vivo* model, imipramine treatment reduced viral loads and alleviated clinical signs in FIPV-infected cats. These findings collectively identify imipramine as a promising, host-targeted therapeutic candidate for FIP.

## INTRODUCTION

Feline coronaviruses (FCoVs) are single-stranded, positive-sense RNA viruses belonging to the genus *Alphacoronavirus* within the family *Coronaviridae* of the order *Nidovirales* and are widespread pathogens among both domestic and wild felids (1). Based on their pathogenicity, FCoVs are classified into two biotypes: feline enteric coronavirus (FECV) and feline infectious peritonitis virus (FIPV). Cats infected with FECV are typically asymptomatic or exhibit only mild, self-limiting gastrointestinal inflammation, and most infected animals eventually clear the virus (2). FIPV is widely believed to arise from spontaneous mutations of FECV, usually within monocytes or macrophages, enabling the virus to escape intestinal confinement and disseminate systemically, leading to the development of feline infectious peritonitis (FIP), a fatal immune-mediated disease (3). Due to the phenomenon of antibody-dependent enhancement (ADE), the development of effective FIPV vaccines has been proven extremely challenging (4). Thus, the identification and development of novel antiviral agents targeting FIPV are paramount for the prophylaxis and treatment of FIP.

Imipramine, a classical tricyclic antidepressant approved by the U.S. Food and Drug Administration (FDA), has been widely used since the 1960s for the treatment of psychiatric disorders such as depression and anxiety (5). Like many other antidepressants, imipramine is classified as an amphiphilic cationic drug (CAD), a class of compounds that selectively accumulate within intracellular acidic organelles, including endosomes and lysosomes. This selective enrichment perturbs cholesterol metabolism, lipid homeostasis, and the integrity of intracellular membrane systems (6). These mechanisms are not only central to its antidepressant activity but also underlie its emerging potential in antiviral therapy. It has been shown that imipramine can interfere with viral entry, uncoating, or intracellular trafficking by modulating the endosomal-lysosomal pathway. Imipramine exhibited broad-spectrum antiviral activity against various viruses, including chikungunya virus, bluetongue virus, Ebola virus, and Zika virus (7, 8). Given its capacity to perturb host lipid metabolism and intracellular membrane integrity, imipramine is increasingly recognized as a promising candidate for antiviral drug repurposing—especially for emerging or re-emerging viruses for which effective therapies are currently lacking.

The antiviral potential of imipramine is closely associated with its function as an inhibitor of Niemann-Pick C1 (NPC1), a transmembrane transporter protein localized on endosomal and lysosomal membranes (9, 10). NPC1 is essential for maintaining cellular cholesterol homeostasis (11), working in concert with the soluble luminal protein NPC2 to mediate the trafficking of cholesterol derived from endocytosed low-density lipoprotein (LDL) from lysosomes to other cellular compartments, notably the plasma membrane and endoplasmic reticulum (12). The human NPC1 protein comprises 1,278 amino acids, and its N-terminal domain (NTD) specifically binds cholesterol to facilitate its transmembrane transport (13). Beyond its metabolic functions, NPC1 plays a pivotal role in the life cycle of several viruses. For example, Ebola virus requires a direct interaction between its glycoprotein and NPC1 to mediate the fusion of viral and endosomal membranes, enabling the release of the viral genome into the host cytoplasm (14). Similar NPC1 dependency has been reported for Zika virus, SARS-CoV-2, and other enveloped viruses (15), positioning NPC1 as a promising broad-spectrum antiviral host target. However, the exact role of NPC1 in the context of FIPV infection remains unclear, warranting further investigation.

This study systematically evaluates the antiviral activity of imipramine against FIPV. We found that imipramine potently inhibited FIPV replication *in vitro* and *in vivo*. Treatment during viral entry, infection, or post-entry stages was effective, with co-treatment yielding the strongest suppression. Mechanistically, imipramine disrupted both viral entry and replication independently of type I interferon signaling and also attenuated the FIPV-induced expression of pro-inflammatory cytokines like IL-6 and TNF-α. These results unveil a multitargeted antiviral mechanism for imipramine and support its repurposing as a promising treatment for feline infectious peritonitis.

## MATERIALS AND METHODS

### Cells and virus

Crandell-Rees feline kidney (CRFK) cells (ATCC CCL-94) were cultured in Dulbecco’s modified Eagle medium (DMEM; Gibco, USA) supplemented with 10% fetal bovine serum (FBS; Gibco, USA) and 1% penicillin-streptomycin (Gibco, USA) at 37°C in a humidified incubator containing 5% CO_2_. The DF-2 strain of FIPV (GenBank accession no. JQ408981.1), which is capable of infecting CRFK cells, was stored at –80°C and thawed immediately prior to use.

### Reagents and antibodies

Imipramine HCl (S4344, Selleck, USA) was dissolved in DMSO to make a 12.5 mM stock. Stocks were stored at –80°C protected from light. Working concentrations were prepared by diluting stocks in serum-free DMEM. A rabbit polyclonal antibody against the FIPV nucleocapsid (N) protein was prepared in our laboratory and stored at –80°C. The Alexa Fluor 488 donkey anti-rabbit IgG antibody was purchased from Thermo Fisher Scientific (USA).

### Virus infection and chemical treatment

CRFK cells were infected with FIPV (MOI = 0.01) or mock-infected with DMEM. To enhance viral entry, 5 μg/mL trypsin (0.25%, Gibco, USA) was added during infection. The virus-cell mixture was incubated at 37°C for 2 h. After incubation, cells were washed three times with serum-free DMEM to remove unbound virus particles. Both infected and mock-infected groups were then supplied with DMEM containing 2.5 μg/mL trypsin and incubated at 37°C until sample collection.

For drug treatment, CRFK cells were seeded into 24-well plates 12 h prior to infection. Cells were pretreated with imipramine at the indicated concentrations for 1 h at 37°C, based on a previously established protocol. After pretreatment, cells were infected with FIPV for 2 h. Following infection, the culture medium was replaced with serum-free DMEM containing the corresponding drug concentration, and the cells were incubated until the indicated time points for analysis.

### Cell viability assay and determination of 50% cytotoxicity (CC_50_)

Cellular toxicity of the drugs was assessed using the CellTiter-Glo Luminescent Cell Viability Assay Kit (Promega, USA). CRFK cells were seeded in 96-well plates and cultured overnight to reach full confluence, and then the culture medium was removed and replaced with varying concentrations of the test compounds. Each concentration was tested in quadruplicate. After 24 h of incubation at 37°C, the supernatant was discarded and replaced with a 1:1 mixture of CellTiter-Glo reagent and serum-free DMEM. The plates were shaken for 5 min to ensure complete cell lysis, followed by a 10-min incubation at room temperature to stabilize the luminescent signal. Luminescence was recorded, and cytotoxicity was calculated using the following formula (16): Cytotoxicity (%) = [1-luminescence values (drug treatment-blank)/luminescence values (control-blank)] × 100%.

The concentration of each compound that induced CC_50_ was determined using nonlinear regression analysis in GraphPad Prism 8.

### RNA extraction, quantitative real-time PCR (RT-qPCR), and determination of half-maximal effective concentration (EC_50_)

Total RNA was extracted from cells at the indicated time points post-infection using the VAMNE Magnetic Cell/Tissue Total RNA Kit (Prepackaged) (Vazyme, China) via a fully automated RNA extraction system (Vazyme, China). Subsequently, 1 μg of total RNA was reverse-transcribed into cDNA using the HiScript IV All-in-One Ultra RT SuperMix for qPCR (Vazyme, China).

RT-qPCR was conducted on the LightCycler 480 II system (Roche, Switzerland) using Taq Pro Universal SYBR qPCR Master Mix (Vazyme, China). Gene-specific primers are listed in Table 1. Relative expression levels of target genes were normalized to the internal reference gene *GAPDH* and calculated using the 2^-ΔΔCT^ method. The inhibition rate of FIPV replication at each drug concentration was determined using the following formula (16): Inhibition (%) = (1-relative expression of viral RNA in drug treatment/expression of viral RNA in virus control) × 100%.

**TABLE 1:** Sequences of primers used for RT-qPCR

Primer name	Sequence (5′−3′)
GAPDH-qPCR-F	TGACCACAGTCCATGCCATC
GAPDH-qPCR-R	GCCAGTGAGCTTCCCATTC
FIPV-qPCR-F	GATTTGATTTGGCAATGCTAGATTT
FIPV-qPCR-R	AACAATCACTAGATCCAGACGTTAGCT
NPC1-qPCR-F	GTGACGAGTCTGTGGACGAG
NPC1-qPCR-R	CTCCCCTCTGTCATTGGCAT
IFN-β-qPCR-F	GAAGGAGGAAGCCATATTGGT
IFN-β-qPCR-R	CTCCATGATTTCCTCCAGGAT
Mx1-qPCR-F	TTCGGAGGTGGAGGAGGCAATC
Mx1-qPCR-R	CAGGGAGGTCTATCAGGGTCAGATC
IFIT3-qPCR-F	TGAAGCTGGCAAGAATGGAGAGAAG
IFIT3-qPCR-R	GGAGGTCGGTGACATCAGAATATGC
IL-6-qPCR-F	TGCCTGACAAGAATCACTACT
IL-6-qPCR-R	GAACTACAGCAATCTTAGATG
TNF-α-qPCR-F	CTTCTCGAACTCCGAGTGACAAG
TNF-α-qPCR-R	CCACTGGAGTTGCCCTTCA

The EC_50_ was calculated using GraphPad Prism 8.

### Immunofluorescence assay (IFA)

The effect of the compounds on viral infection was assessed using an IFA. Cells subjected to various experimental conditions were first fixed with 4% paraformaldehyde at 4°C for 30 min in the dark. After three washes with PBS (Solarbio, China), the cells were permeabilized with 0.2% Triton X-100 for 20 min at room temperature, followed by another PBS wash. This was followed by blocking with PBS containing 5% FBS and 5% skim milk (Sigma-Aldrich, USA) at 37°C for 1 h. Cells were then incubated with rabbit anti-FIPV N protein polyclonal antibody (1:2,000) at 37°C for 1.5 h, followed by incubation with Alexa Fluor 488 donkey anti-rabbit IgG antibody (1:1,000) at 37°C for 1 h. After three additional PBS washes, nuclear DNA was counterstained with 4′,6-diamidino-2-phenylindole (DAPI) for 10 min at room temperature. Finally, cells were visualized using a Nikon Ti2-E fluorescence microscope.

### Viral titration assay

Viral titers were determined by the 50% tissue culture infectious dose (TCID_50_) assay. CRFK cells were seeded in 96-well plates in advance and allowed to reach full confluence. Serial 10-fold dilutions of viral supernatants (from 10^−1^ to 10^−8^) were prepared, and 8 replicate wells were used for each dilution. Following incubation at 37°C for 3–4 days, cytopathic effects (CPE) were observed and recorded under an inverted microscope. Viral titers were calculated using the Reed-Muench method and expressed as TCID_50_.

### Time-of-addition assay

To determine the stage of the FIPV life cycle at which imipramine exerts its antiviral effects, a time-of-addition assay was performed based on a modified version of a previously established protocol in our laboratory (17).

For the virus binding assay, CRFK cells were first incubated with imipramine or DMSO at 37°C for 1 h. After removing the supernatant, pre-chilled FIPV (MOI = 10) was added, and the cells were incubated at 4°C for 1 h to allow virus binding. Unbound virus was removed by washing with cold PBS, and total RNA was then extracted from the cells.

For the virus entry assay, cells were first incubated with FIPV (MOI = 10) at 4°C for 1 h to allow binding. After washing three times with cold PBS to remove unbound virus, serum-free DMEM containing imipramine or DMSO was added. The cells were then incubated at 37°C for 2 h to permit viral entry. Subsequently, uninternalized virus was removed by washing three times with citrate buffer (40 mM citric acid, 10 mM KCl, and 135 mM NaCl, at pH = 3.0), followed by three washes with PBS to remove residual acid. RNA was then extracted for analysis.

For the viral replication assay, cells were infected with FIPV (MOI = 0.01) for 2 h, after which imipramine or DMSO was added. At 24 h post-infection (hpi), both cells and supernatants were collected to evaluate whether imipramine affected the replication phase of the virus.

### Gene knockdown assay

To investigate the effect of NPC1 knockdown on FIPV infection, synthesized siRNA targeting the feline *NPC1* gene (siRNA sequences are listed in Table 2) and a negative control (NC) siRNA were transfected into CRFK cells, followed by viral infection. CRFK cells were seeded in 24-well plates in advance, and transfection was initiated when cell confluency reached approximately 80%. Prior to transfection, siRNA or NC and Lipofectamine RNAimax transfection reagent (Invitrogen, USA) were each diluted in Opti-MEM (Gibco, USA) and incubated separately at room temperature for 20 min. The two solutions were then combined to prepare the transfection mixture. The culture medium was replaced with serum-free DMEM before transfection. The transfection mixture was gently added dropwise to each well and incubated at 37°C for 6–8 h. After transfection, the supernatant was removed and replaced with complete DMEM for further incubation.

**TABLE 2:** siRNA sequences targeting NPC1

siRNA name	Sequence (5′−3′)
NC-sense	UUCUCCGAACGUGUCACGUTT
NC-antisense	ACGUGACACGUUCGGAGAATT
156-sense	GAAGGAUGGGUAUGACUUATT
156-antisense	UAAGUCAUACCCAUCCUUCTT
1166-sense	GCCUCGAGAAAGAGUACUUTT
1166-antisense	AAGUACUCUUUCUCGAGGCTT
1485-sense	GGACCAUGAAAUAGGAGAUTT
1485-antisense	AUCUCCUAUUUCAUGGUCCTT

### Cellular cholesterol detection assay

The experimental protocol was adapted and optimized from a previously published method (18). The procedure closely followed that of the immunofluorescence assay, with the exception that DAPI staining was replaced by staining with 0.5 mg/mL Filipin complex (MedChemExpress, USA), which specifically binds to unesterified cholesterol. After incubation at room temperature for 3 h in the dark, cells were washed three times with PBS. Intracellular cholesterol distribution was then visualized using a fluorescence microscope.

### Pseudovirus assay

The pseudovirus of the FIPV DF-2 strain was constructed in our laboratory and stored at −80°C. CRFK cells were seeded in 96-well plates in advance. Once the cells reached full confluence, they were treated with imipramine or DMSO and incubated at 37°C for 1 h. After removing the supernatant, a mixture of pseudovirus or control vector diluted in DMEM was added, followed by incubation at 37°C for 16 h. Subsequently, the supernatant was discarded and replaced with a 1:1 mixture of Steady-Glo Luciferase Assay System (Promega, USA) and serum-free DMEM. After shaking for 5 min to ensure complete cell lysis, luciferase activity was measured using the Varioskan ALF system (Thermo Fisher, USA).

### Animal infection and treatment

Nine healthy 2-month-old female cats were randomly divided into three groups (*n* = 3/group): mock, FIPV, and FIPV+imipramine. Each group was housed in a separate isolated cage. On day 0, cats in the FIPV and FIPV+imipramine groups were intraperitoneally inoculated with 1 × 10^6^ TCID_50_ of FIPV DF-2, while mock cats received the same volume of DMEM. From day 0 to day 14, the FIPV+imipramine group was treated twice daily with imipramine (5 mg/kg), whereas mock and FIPV groups received equal volumes of saline. Body weight and rectal temperature were recorded daily. Blood samples were collected on days 0, 7, and 14 for hematology and serum biochemistry. On day 14, all cats were anesthetized with propofol and chloral hydrate, euthanized, necropsied, and samples were collected for viral load determination.

### Statistical analysis

All experiments in this study were independently repeated for at least three independent biological replicates, with each biological replicate being performed in triplicate for technical repeats. Experimental data were analyzed using GraphPad Prism 10.1.2 (GraphPad Software, San Diego, CA). Statistical significance was assessed using Student’s *t*-test, with significance levels indicated as follows: *, *P <* 0.05; **, *P <* 0.01; ***, *P <* 0.001; ****, *P <* 0.0001; and ns (not significant).

## RESULTS

### Imipramine dose-dependently inhibits FIPV infection

**Fig 1:**
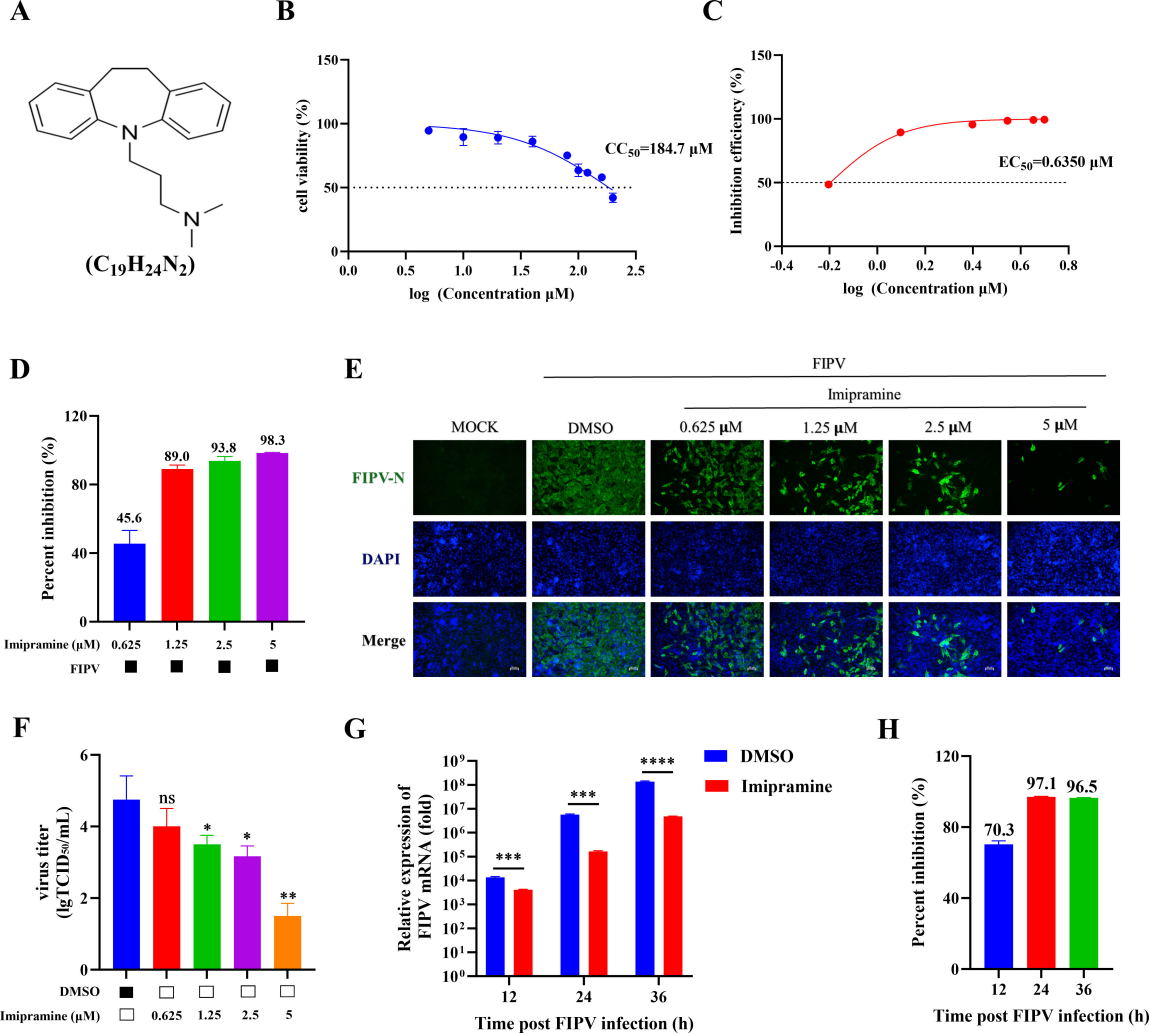
Imipramine dose-dependently inhibits FIPV infection. (**A**) Chemical structure of imipramine. (**B**) Effect of imipramine on CRFK cell viability. Cells were treated with varying concentrations of imipramine for 24 h, and the viability was assessed using the CellTiter-Glo Luminescent Cell Viability Assay. The half-maximal cytotoxic concentration (CC_50_) was calculated. (**C**) Inhibitory curve of FIPV infection by imipramine. CRFK cells were infected with FIPV and treated with different concentrations of imipramine for 1 h. The same concentrations were maintained post-infection, and viral RNA levels were quantified by RT-qPCR at 24 hpi. The inhibition rate at each concentration was calculated and used to determine the half-maximal effective concentration (EC_50_). (**D–F**) Cells were pretreated with 0.625, 1.25, 2.5, or 5 μM imipramine for 1 h, followed by FIPV infection (MOI = 0.01) and continued exposure to the same drug concentrations. After 24 h, viral RNA levels were quantified by RT-qPCR (**D**), N protein expression was visualized by immunofluorescence (**E**), and viral titers in the supernatant were determined by TCID_50_ assay (**F**). (**G–H**) Time-course analysis of antiviral activity. Cells were treated with DMSO or 5 μM imipramine for 1 h, then infected with FIPV (MOI = 0.01), and maintained under the same drug concentration post-infection. Cells were harvested at 12, 24, and 36 hpi for viral RNA quantification by RT-qPCR. Viral RNA levels in both control and treatment groups are shown (**G**), and the inhibition rate at each time point is presented (**H**). Data are presented as mean ± standard deviation (SD) from three independent experiments. Statistical analysis was performed using Student’s *t*-test. * *P* < 0.05; **P < 0.01; ***P < 0.001; ****P < 0.0001; ns, not significant.

Imipramine, a synthetic tricyclic antidepressant, has the molecular structure shown in Fig. 1A. We initially assessed its cytotoxicity in CRFK cells after treatment with serial concentrations of imipramine. The half-maximal cytotoxic concentration (CC_50_) was 184.7 μM (Fig. 1B), indicating low cytotoxicity within the tested range and a favorable safety profile.

To assess imipramine’s antiviral activity against FIPV, CRFK cells were pretreated with various concentrations of imipramine or DMSO for 1 h and then infected with FIPV (MOI = 0.01) for 24 h. RT-qPCR showed that imipramine significantly inhibits FIPV infection in a concentration-dependent manner (EC_50_ = 0.635 µM; Fig. 1C) with a high selectivity index (SI = CC_50_/EC_50_= 291). Notably, 5 μM imipramine inhibited FIPV infection by 98.3% (Fig. 1D). The potent anti-FIPV activity of imipramine was further confirmed by IFA targeting FIPV-N protein (Fig. 1E). Consistently, the TCID_50_ assay of supernatants demonstrated decreased viral titers post-imipramine treatment, indicating inhibition of the production of functional viral particles (Fig. 1F). Viral growth kinetics were determined by measuring viral RNA levels at 12, 24, and 36 hpi in CRFK cells (Fig. 1G). Compared with the DMSO-treated control group, imipramine markedly suppressed viral replication at all time points, resulting in inhibition rates of 70.3%, 97.1%, and 96.5% at 12, 24, and 36 hpi, respectively, with peak suppression observed at 24 hpi (Fig. 1H). Together, these data indicate imipramine exhibits potent dose-dependent antiviral activity against FIPV.

### Imipramine inhibits FIPV infection across regimens

**Fig 2:**
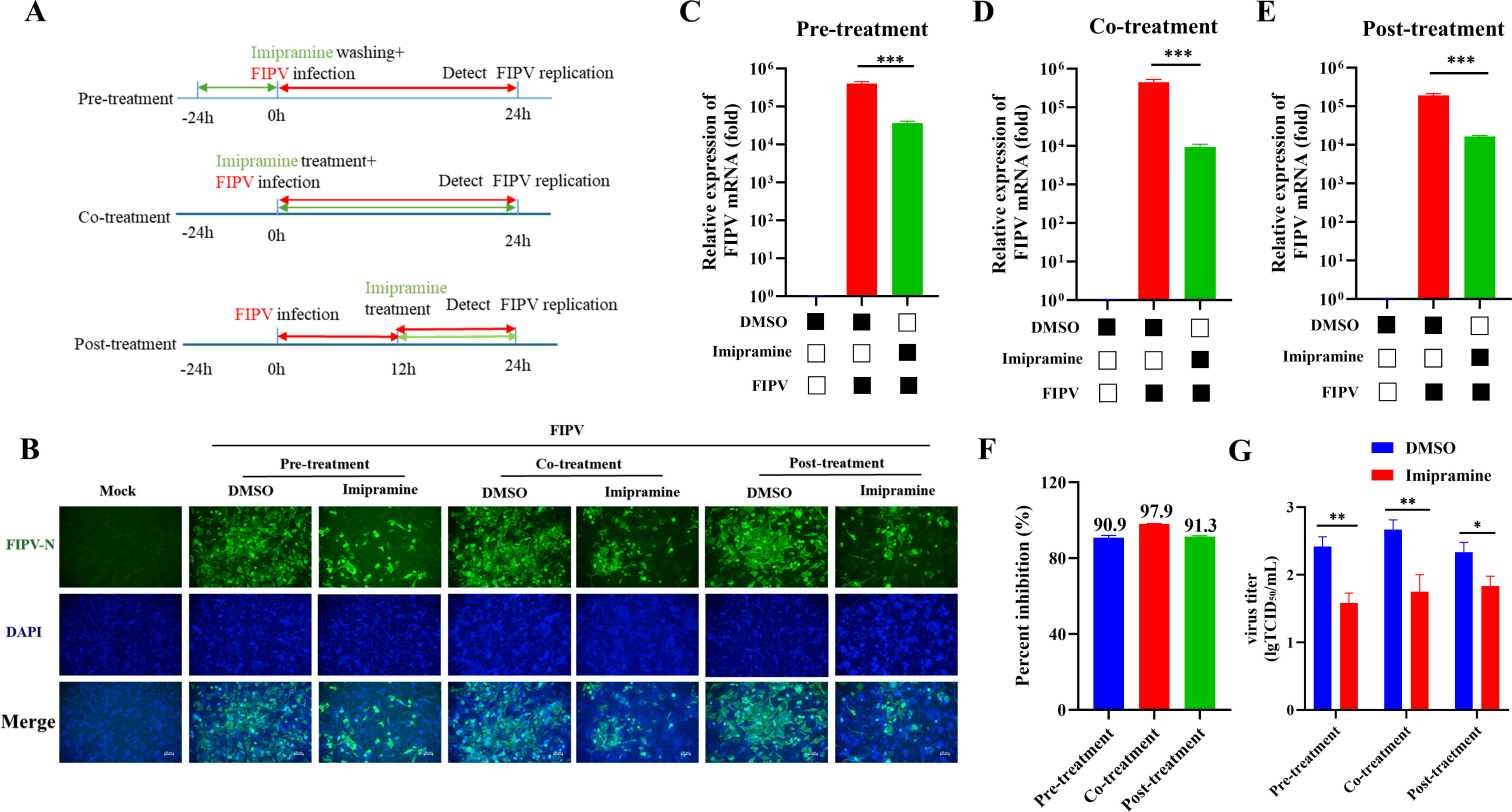
Imipramine inhibits FIPV infection across regimens. (**A**) Schematic representation of imipramine treatment strategies. Pretreatment: CRFK cells were pretreated with 5 μM imipramine or DMSO for 24 h prior to FIPV infection. Co-treatment: Cells were simultaneously treated with 5 μM imipramine or DMSO at the time of FIPV infection (MOI = 0.01). Post-treatment: Cells were treated with 5 μM imipramine or DMSO at 12 hpi. (**B–F**) Cells from each treatment group were collected at 24 hpi. FIPV infection was assessed by immunofluorescence staining for viral N protein (**B**) and by RT-qPCR for viral RNA levels (**C–E**). The inhibition rate of FIPV infection for each treatment condition was calculated (**F**). (**G**) Viral titers in the culture supernatants were determined by TCID_50_ assay. Data are presented as the mean ± standard deviation (SD) from three independent experiments. Statistical significance was determined using Student’s *t*-test. * *P* < 0.05; **P < 0.01; *** *P* < 0.001; ****P < 0.0001; ns, not significant.

To explore imipramine’s stage-specific efficacy against FIPV replication, we tested imipramine or DMSO in CRFK cells at pre-, co-, or post-viral exposure (Fig. 2A). IFA revealed a marked reduction in FIPV N protein expression across all regimens (Fig. 2B). Consistently, RT-qPCR revealed that co-treatment most potently suppressed viral genomes (97.9% reduction), followed by pretreatment (90.9% reduction) and post-infection treatment (91.3% reduction) (Fig. 2C through F). TCID_50_ assay of supernatants confirmed this trend (Fig. 2G). Together, these data indicate imipramine exhibits significant antiviral activity against FIPV irrespective of the treatment timing, with co-treatment most effective.

### Imipramine inhibits FIPV entry and replication

**Fig 3:**
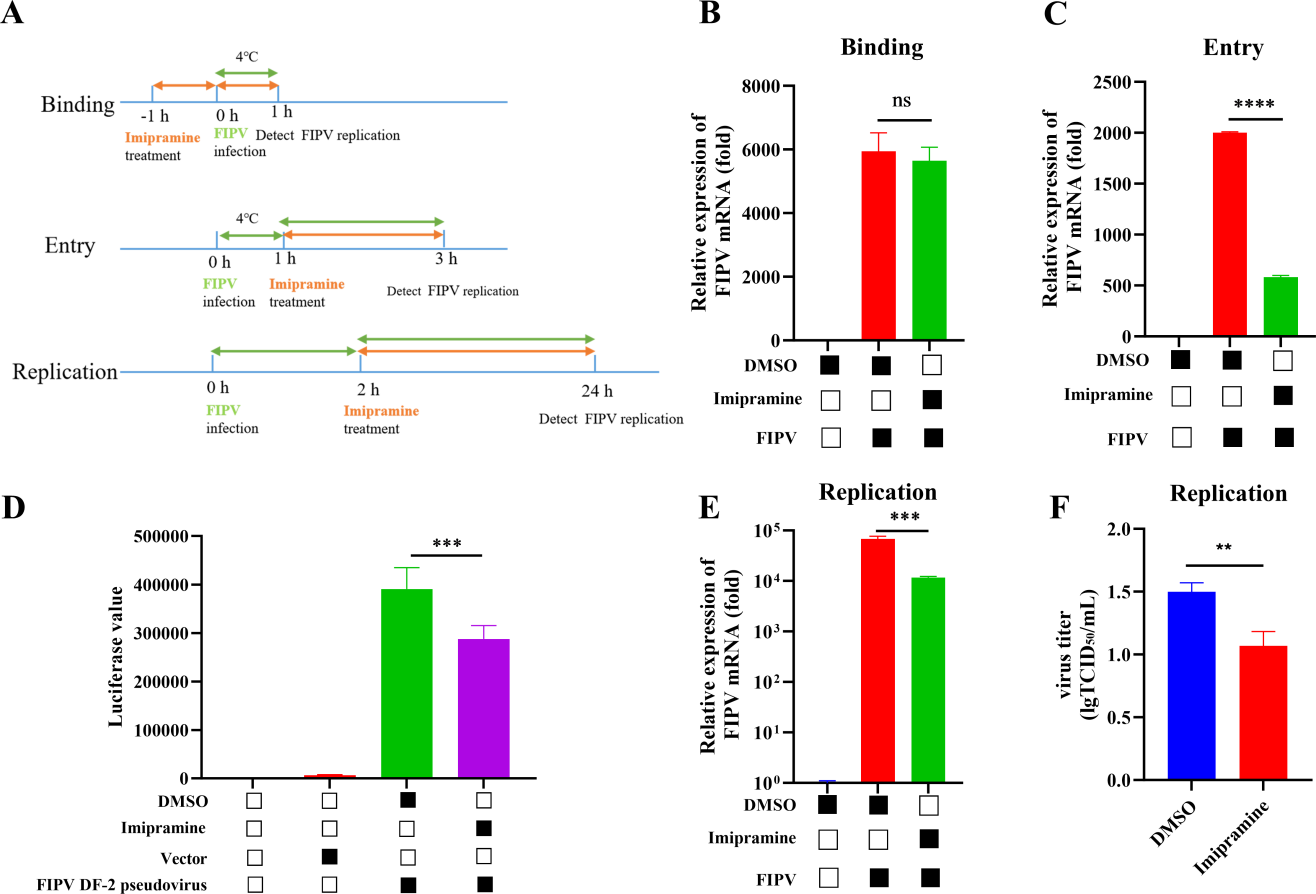
Imipramine inhibits FIPV entry and replication. (**A**) Schematic diagram of the time-of-addition assay. (**B**) CRFK cells were treated with 5 μM imipramine or DMSO for 1 h at 37°C, followed by infection with FIPV (MOI = 10) for 1 h at 4°C to allow viral binding. Cells were then washed three times with pre-cooled PBS and collected, and FIPV RNA levels were quantified by RT-qPCR. (**C**) After virus binding at 4°C for 1 h, cells were incubated with 5 μM imipramine or DMSO and shifted to 37°C for 2 h to allow viral internalization. Cells were then washed three times with citrate buffer and three times with PBS to remove uninternalized viruses. Intracellular viral RNA was measured by RT-qPCR. (**D**) CRFK cells were pretreated with 5 μM imipramine or DMSO for 1 h, followed by infection with pseudovirus of FIPV DF-2 strain or vector control. After 16 h, luciferase activity was measured in each well. (**E–F**) CRFK cells were infected with FIPV, and 5 μM imipramine or DMSO was added at 2 hpi. At 24 hpi, viral RNA expression was assessed by RT-qPCR (**E**), and viral titers in the supernatants were determined by TCID_50_ assay (**F**). Data are presented as the mean ± standard deviation (SD) from three independent experiments. Statistical significance was determined using Student’s *t*-test. * *P* < 0.05; **P < 0.01; ***P < 0.001; **** *P* < 0.0001; ns, not significant.

To further identify the specific stages of the FIPV life cycle targeted by imipramine, we performed time-of-addition assays to assess viral binding, entry, and replication (Fig. 3A). RT-qPCR results revealed that imipramine had no significant effect on viral binding (Fig. 3B) but strongly inhibited entry (Fig. 3C), identifying this as its primary target. This finding was confirmed using a luciferase-expressing FIPV pseudovirus in a single-round infection assay, which showed reduced entry efficiency upon imipramine treatment (Fig. 3D). Furthermore, post-entry treatment with imipramine reduced both intracellular viral RNA and extracellular viral titers (Fig. 3E and F), indicating a secondary effect on viral replication. Together, these results demonstrate that imipramine targets multiple stages of the FIPV life cycle, primarily entry and secondarily replication.

### Imipramine exerts antiviral activity by inhibiting NPC1 function

**Fig 4:**
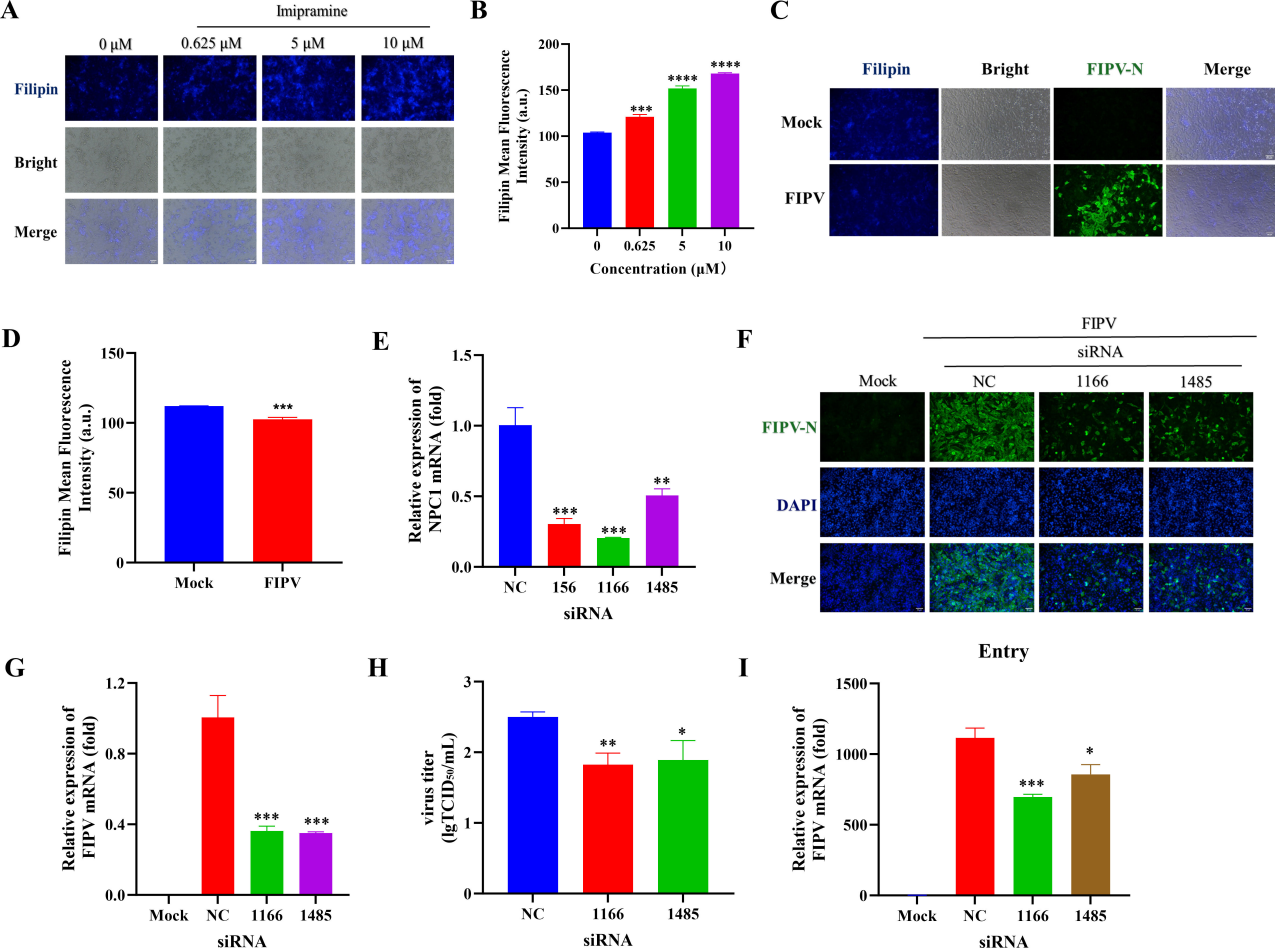
Imipramine exerts antiviral activity by inhibiting NPC1 function. (**A–B**) CRFK cells were treated with different concentrations of imipramine (0.625, 5, and 10 μM) for 24 h. Cells were collected, and intracellular cholesterol levels were measured. Representative Filipin staining images are shown in **A**, and the fluorescence intensity was quantified using ImageJ (**B**). (**C–D**) CRFK cells were infected with FIPV (MOI = 10) and collected at 24 hpi for immunofluorescence staining of FIPV N protein and quantification of cellular cholesterol levels. Representative Filipin staining images are shown in **C**, and fluorescence intensity was quantified using ImageJ (**D**). (**E**) CRFK cells were transfected with NPC1-specific siRNA or negative control (NC). After 24 h, cells were harvested, and NPC1 mRNA expression was assessed by RT-qPCR to confirm knockdown efficiency. (**F–H**) CRFK cells transfected with NPC1 siRNA or NC were infected with FIPV (MOI = 0.01). At 24 hpi, cells were fixed for immunofluorescence staining of FIPV N protein (**F**), FIPV RNA levels were quantified by RT-qPCR (**G**), and viral titers in the supernatants were determined using TCID_50_ assay (**H**). (**I**) CRFK cells transfected with NPC1 siRNA or NC were infected with FIPV (MOI = 10) after 24 h. After 1 h of viral binding at 4°C, cells were washed three times with pre-cooled PBS to remove unbound virus and then incubated at 37°C for 2 h to allow viral internalization. Cells were subsequently washed three times with citrate buffer and three times with PBS to eliminate the residual acid. The amount of internalized viruses was determined by RT-qPCR. All results are presented as the mean ± standard deviation (SD) of three independent experiments. Statistical significance was assessed using Student’s *t*-test. * *P* < 0.05; **P < 0.01; ***P < 0.001; ****P < 0.0001; ns, not significant.

Given that imipramine disrupts FIPV entry and is a known modulator of lipid metabolism and NPC1 function (7), we hypothesized that its antiviral activity involves the disruption of NPC1-mediated cholesterol trafficking. To test this, we first treated CRFK cells with imipramine and observed intracellular cholesterol accumulation via Filipin staining (Fig. 4A and B), confirming NPC1 dysfunction. We then demonstrated that FIPV infection itself reduces intracellular cholesterol (Fig. 4C and D), suggesting the virus exploits NPC1-mediated trafficking. To directly link NPC1 function to infection, we performed an NPC1 siRNA knockdown (Fig. 4E), which significantly reduced FIPV N protein expression (Fig. 4F), viral RNA (Fig. 4G), and supernatant viral titers (Fig. 4H). Furthermore, viral entry assays confirmed that NPC1 knockdown impairs FIPV entry (Fig. 4I), mirroring the effect of imipramine. Together, these results establish NPC1 as critical for FIPV entry and demonstrate that imipramine exerts its antiviral effect, at least in part, by impairing NPC1 function.

### Imipramine inhibits FIPV-induced upregulation of inflammatory cytokines

**Fig 5:**
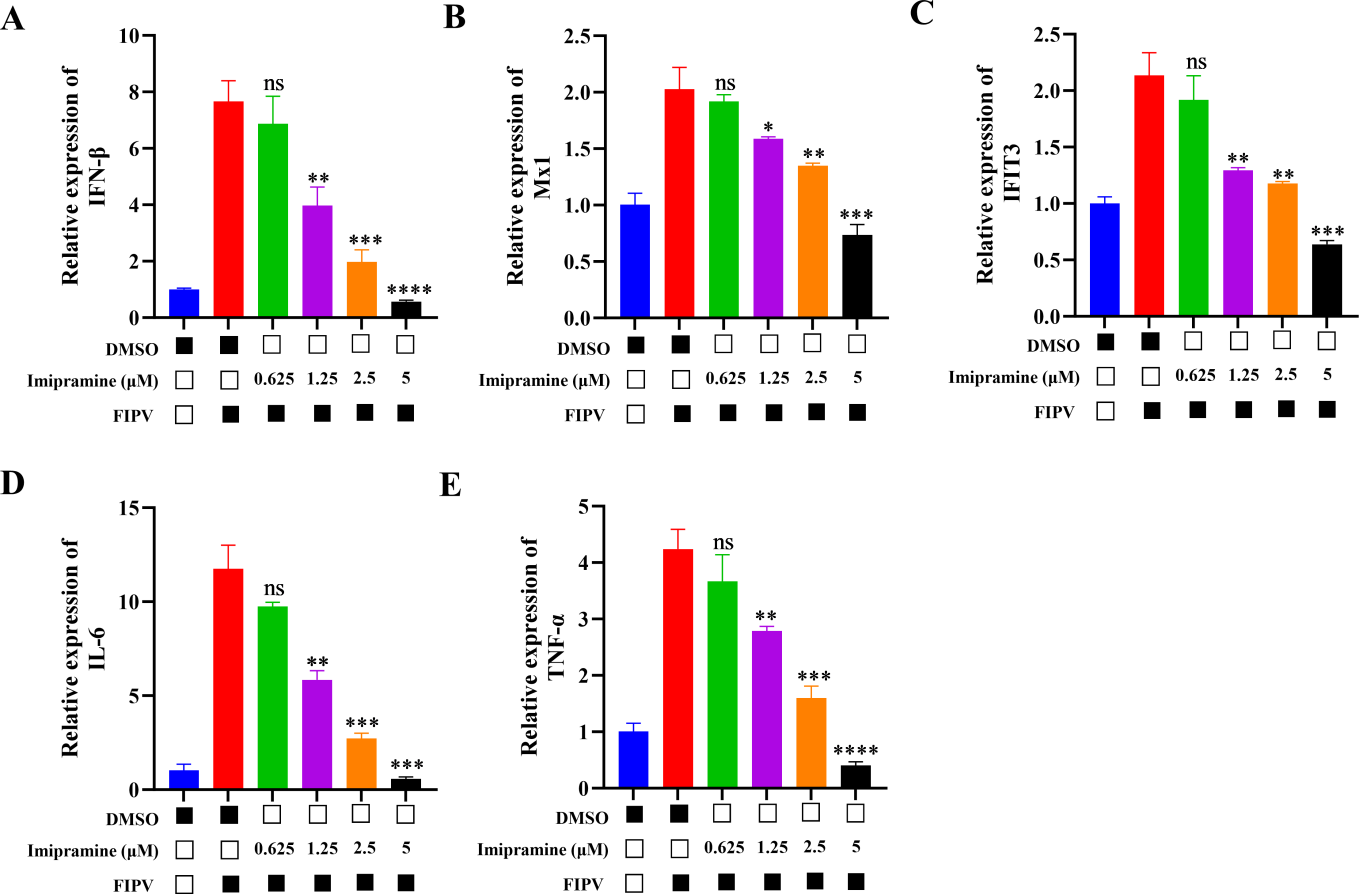
Imipramine inhibits FIPV-induced upregulation of inflammatory cytokines. (**A–E**) CRFK cells were pretreated with various concentrations of imipramine or DMSO for 1 h, followed by infection with FIPV (MOI = 0.01). Cells were maintained in the corresponding drug concentrations after infection and harvested at 24 hpi. The mRNA expression levels of IFN-β (**A**), Mx1 (**B**), IFIT3 (**C**), IL-6 (**D**), and TNF-α (**E**) were quantified by RT-qPCR. Data are presented as the mean ± standard deviation (SD) of three independent experiments. Statistical analysis was performed using Student’s *t*-test. *P < 0.05; ** *P* < 0.01; *** *P* < 0.001; ****P < 0.0001; ns, not significant.

The primary clinicopathological features of FIPV infection—such as fever, vasculitis, and serositis—are driven by a pro-inflammatory state and cytokine release (19). Given the established role of cholesterol metabolism in regulating inflammation (20), we investigated whether imipramine, an NPC1 inhibitor, could modulate these virus-elicited inflammatory responses beyond direct antiviral activity. Given the critical role of type I IFN (IFN-α/β) in antiviral immunity (21), we initially evaluated whether imipramine acts via the type I IFN pathway. We assessed imipramine’s effect on IFN-β and interferon-stimulated genes (ISGs) Mx1 and IFIT3 to test direct type I IFN modulation. However, imipramine treatment did not upregulate IFN-β (Fig. 5A) nor alter the expression of Mx1 and IFIT3 (Fig. 5B and C), indicating its antiviral mechanism is type I IFN-independent. We then assessed its effect on key inflammatory cytokines. Consistent with FIPV-induced cytokine storms, infection strongly elevated IL-6 and TNF-α. Imipramine treatment significantly suppressed this induction (Fig. 5D and E), revealing a potent anti-inflammatory activity. In conclusion, imipramine suppresses FIPV through type I IFN-independent mechanisms, and its therapeutic benefits are likely augmented by the direct attenuation of virus-induced inflammation, supporting its utility as a multitargeted therapeutic for FIP.

### Imipramine significantly inhibits FIPV infection *in vivo*

**Fig 6:**
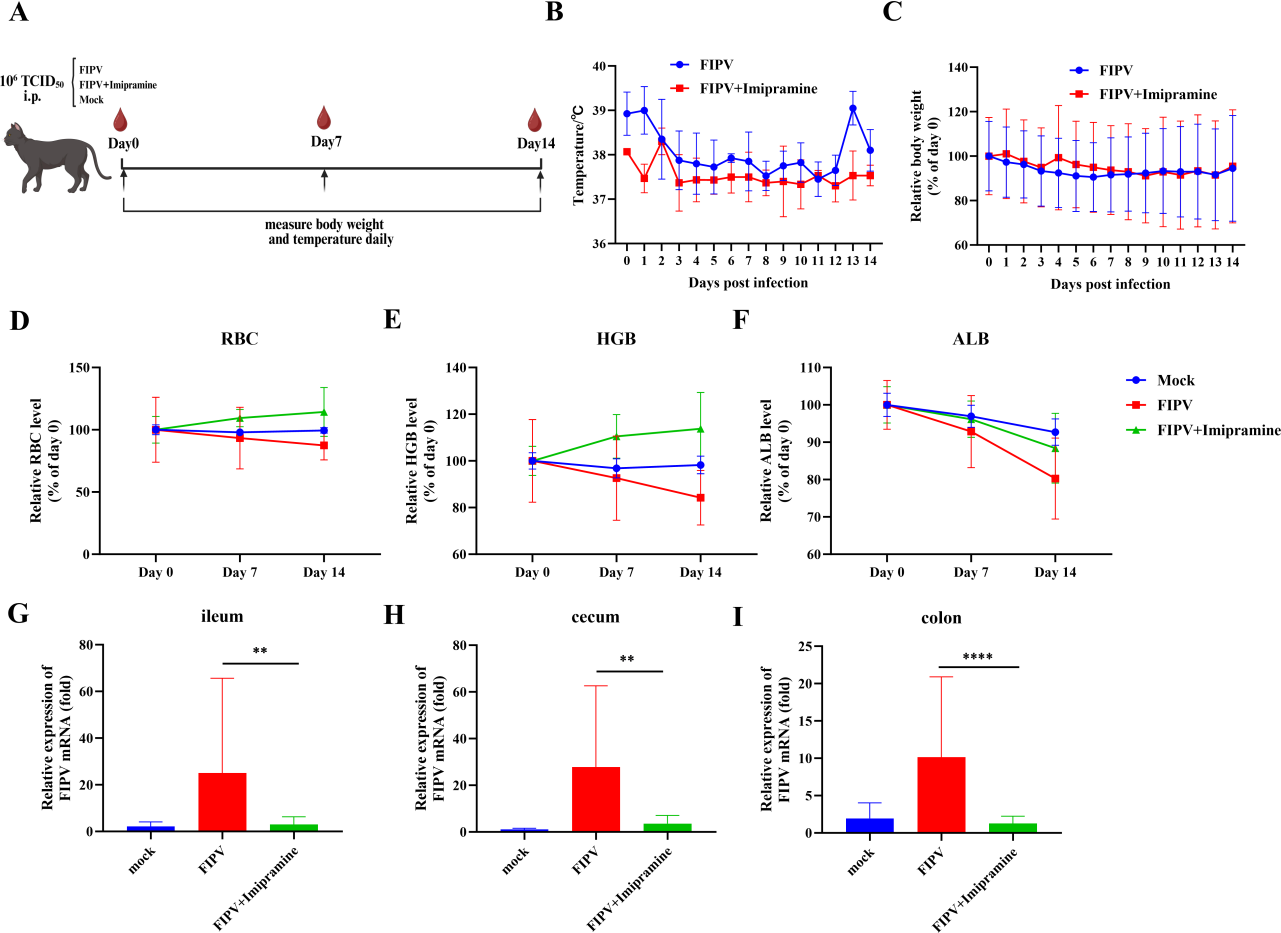
Imipramine significantly inhibits FIPV infection *in vivo.* (**A**) Schematic diagram of the animal experimental design. Nine healthy 2-month-old female cats were randomly divided into three groups (mock, FIPV, and FIPV+imipramine) and monitored for 14 days. (**B**) Changes in body temperature of cats in the FIPV and FIPV+imipramine groups during the 14-day observation period. (**C**) Changes in body weight of cats in the FIPV and FIPV+imipramine groups during the 14-day observation period. (**D–F**) Hematological and biochemical parameters, including red blood cell count (RBC) (**D**), hemoglobin (HGB) (**E**), and albumin (ALB) (**F**), measured on days 0, 7, and 14. (**G–I**) Viral loads in the ileum (**G**), cecum (**H**), and colon (**I**) of cats from each group on day 14. Data are presented as mean ± standard deviation (SD). Statistical analysis was performed using Student’s *t*-test. *P < 0.05; ** *P* < 0.01; *** *P* < 0.001; ****P < 0.0001; ns, not significant.

Next, we evaluated the *in vivo* efficacy of imipramine against FIPV (Fig. 6A). Nine 2-month-old healthy female cats were randomized into three groups: mock-infected, FIPV-infected, and FIPV-infected with daily imipramine treatment. Over the 14-day study, we monitored clinical signs and collected serial blood samples for hematology, biochemistry, and viral load analysis. FIPV-infected cats developed persistent fever and progressive weight loss, both of which were markedly alleviated by imipramine treatment (Fig. 6B and C). Hematological and biochemical analyses on days 7 and 14 revealed that infected, untreated cats had significantly reduced hemoglobin (HGB), red blood cell (RBC), and albumin (ALB) levels compared to the imipramine-treated group, indicating more severe anemia and systemic inflammation (Fig. 6D through F). Furthermore, quantification of viral loads demonstrated that imipramine significantly suppressed FIPV replication in the ileum, cecum, and colon (Fig. 6G through I), confirming its potent antiviral activity *in vivo*.

## DISCUSSION

The lack of commercially available treatments or vaccines for FIPV, a fatal feline coronavirus, underscores the urgent need for novel antiviral therapies. In this study, our systematic evaluation demonstrates that imipramine possesses potent, dose-dependent antiviral activity against FIPV with low cytotoxicity, yielding a favorable selectivity index. Time-of-addition and pseudovirus assays revealed that imipramine acts at multiple stages, inhibiting both viral entry and replication. Mechanistically, we found that imipramine suppresses FIPV by inhibiting NPC1 and disrupting cellular cholesterol metabolism.

Imipramine, an NPC1 inhibitor, has demonstrated broad-spectrum antiviral activity in various viral infection models. Previous studies have shown that imipramine can effectively inhibit infections caused by Zika virus, dengue virus, and Ebola virus by disrupting the fusion between the virus and endosomal membranes (8, 22). This effect is primarily mediated by imipramine-induced cholesterol accumulation in the endosome-lysosome system, which compromises membrane integrity, alters endosomal acidity, and blocks viral release, leading to viral retention and loss of infectivity (23). These findings suggest that imipramine exerts its antiviral effects by modulating host cholesterol metabolism and endomembrane homeostasis. Consistent with these observations, our study found that imipramine significantly inhibited FIPV entry, indicating a similar mode of action. Notably, we further demonstrated that imipramine also suppressed viral replication at later stages, a phenomenon not well documented in previous reports. Interestingly, our data showed that the viral replication suppressed by imipramine did not result from the promotion of type I IFN response, and imipramine actually significantly reduced the expression of type I IFNs or their downstream ISGs during FIPV infection. These results indicate that imipramine’s antiviral mechanism is likely independent of the type I IFN pathway and may instead involve disruption of host metabolic pathways or membrane stability essential for viral replication.

The *in vivo* study further confirmed the antiviral efficacy of imipramine observed *in vitro*. Cats treated with imipramine exhibited lower fever intensity, milder weight loss, and improved hematological profiles compared with untreated cats, suggesting that imipramine may reduce systemic inflammation and tissue damage associated with FIPV infection, which is consistent with the reduced expression of IL-6 and TNF-α *in vitro* (Fig. 5D and E). These findings provide physiological evidence supporting its potential as a therapeutic candidate for FIP. Moreover, the reduced viral loads detected in the ileum, cecum, and colon indicate that imipramine effectively suppresses viral replication in tissues, further supporting its *in vivo* antiviral potential. It should be noted that the sample size in this study was limited and the observation period was relatively short. Additional studies with extended treatment durations and different dosage regimens are warranted to fully evaluate the pharmacological and safety profiles of imipramine in cats.

Given the known link between depression and inflammation (24) and the known immunomodulatory properties of the classical antidepressant imipramine, we hypothesized that it might also ameliorate the inflammatory response during FIPV infection. Consistent with this hypothesis, imipramine significantly reduced the expression of the pro-inflammatory cytokines IL-6 and TNF-α following infection. This dual antiviral and anti-inflammatory activity is highly relevant to FIP pathogenesis, which is characterized by both robust viral replication and a destructive host inflammatory response. A therapeutic agent with this multitargeted profile may address the complex pathophysiology of FIP more effectively than a single-target therapy. Collectively, our findings elucidate a multifaceted mechanism of action for imipramine against FIPV and offer novel insights for developing host-targeted antiviral strategies.

### Conclusion

In conclusion, this study demonstrates that imipramine effectively inhibits FIPV infection both *in vitro* and *in vivo* independently of the type I IFN pathway. By suppressing the function of the host NPC1 protein, imipramine interferes with both the viral entry process and the subsequent replication stage. These findings identify imipramine as a promising host-targeted antiviral candidate.

## Data Availability

All data generated or analyzed during this study are included in the article. All reported data required to reanalyze the data are available from the lead contact upon reasonable request.
